# A Case of Paradoxical Cerebral Embolism Due to Pulmonary Arteriovenous Fistula Mimicking Vertebral Artery Dissection With Wallenberg Syndrome

**DOI:** 10.7759/cureus.34564

**Published:** 2023-02-02

**Authors:** Masaru Isogai, Tomoaki Suzuki, Shyunichi Kato, Yoshinori Taniguchi, Hitoshi Hasegawa, Makoto Oishi, Yukihiko Fujii

**Affiliations:** 1 Department of Neurosurgery, Nagaoka Chuo General Hospital, Nagaoka, JPN; 2 Department of Neurosurgery, Niigata University, Brain Research Institute, Niigata, JPN

**Keywords:** hereditary hemorrhagic telangiectasia, double origin of the posterior inferior cerebellar artery, wallenberg syndrome, pulmonary arteriovenous fistula, paradoxical cerebral embolism

## Abstract

Pulmonary arteriovenous fistula (PAVF) leads to paradoxical cerebral embolism, which can be fatal if left untreated. We report a rare case of brainstem infarction with acute severe headache and Wallenberg syndrome caused by a PAVF mimicking vertebral artery (VA) dissection. A 40-year-old man presented with a sudden occipital headache accompanied by right hemisensory disturbance. Magnetic resonance imaging revealed left lateral medullary infarction and poor depiction of the left VA. However, it was clearly recanalized on day six, and there were no findings of VA dissection. Whole-body contrast-enhanced computed tomography (CT) revealed a PAVF in the right lung and a thrombus in the feeding artery. The patient was diagnosed with hereditary hemorrhagic telangiectasia due to recurrent epistaxis and peripheral vasodilation of the tongue. An anticoagulant was administered for preventing further ischemic stroke, and a follow-up CT confirmed the disappearance of the thrombus in three months. Thoracoscopic partial lung resection was performed five months after the onset, and no recurrence of ischemic stroke was observed.

## Introduction

Pulmonary arteriovenous fistula (PAVF) is a condition commonly associated with hereditary hemorrhagic telangiectasia (HHT), and it manifests as a concurrent paradoxical cerebral embolism resulting from a right-to-left shunt [1,2]. If left untreated, the mortality arising from PAVF is relatively high; therefore, early diagnosis is critical [3,4]. The onset of Wallenberg syndrome with headache is often seen in vertebral artery (VA) dissection; however, its manifestation with paradoxical cerebral embolisms of the PAVF is very rare. Here, we present such a rare condition with its investigation, diagnosis, and treatment, along with a discussion of our findings.

## Case presentation

A 40-year-old man presented to a local clinic with primary complaints of sudden occipital headache, right hemisensory disturbance, and dysphagia. These symptoms started two days prior to his arrival at the clinic. The patient’s history included recurrent epistaxis since childhood and lung disease in his 20s. However, the details of his lung disease were unclear, and his family history was also unknown, owing to parental divorce. The local clinic transferred the patient to our hospital due to a suspected cerebral infarction. The patient’s physical examination findings on admission were as follows: height, 176 cm; weight, 57 kg; blood pressure, 129/79 mmHg; heart rate, 67 beats per minute regular; body temperature, 36.5°C; and no impaired consciousness. Along with his right hemisensory disturbance, mild dysarthria and hoarseness were noted.

**Figure 1:**
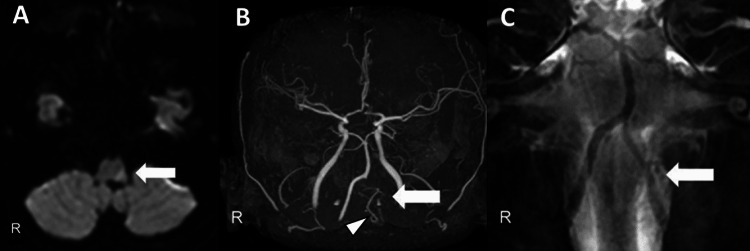
Magnetic resonance images upon hospitalization. (A) Axial image of diffusion-weighted imaging shows hyperintensity in the lateral medulla on the left side (white arrow). (B) Time-of-flight magnetic resonance angiography shows a poor depiction of the left vertebral artery (VA) at the middle segment of V4 (white arrow) and flow signal of the left posterior inferior cerebellar artery (PICA) (white arrowhead). (C) Basi-parallel anatomical scanning shows the V4 segment of the left VA and the bifurcation of the left PICA (white arrow).

Furthermore, the following test findings were noted: hemoglobin (Hb) 15.9 g/dL, D-dimer <0.5 μg/mL, and HbA1c 5.6. Sinus rhythm was observed on 12-lead electrocardiography (ECG). Plain computed tomography (CT) of the head revealed no bleeding or clear low-density areas. Diffusion-weighted imaging (DWI) showed a lesion with a hyperintense area on the left lateral side of the medulla oblongata. Time-of-flight magnetic resonance angiography revealed that the left VA was poorly delineated at the middle segment of V4; however, the left posterior inferior cerebellar artery (PICA) was depicted. Basi-parallel anatomical scanning demonstrated the V4 segment of the left VA and the bifurcation of the left PICA (Figure 1).

**Figure 2:**
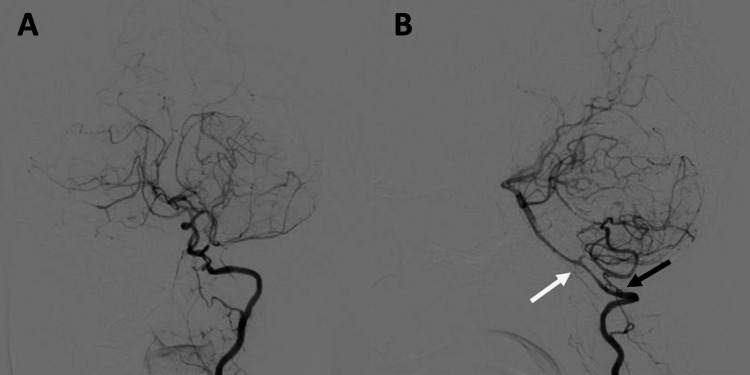
Left vertebral angiography on day six. (A) Anteroposterior view: complete recanalization of the left vertebral artery. (B) Lateral view: double origin of the posterior inferior cerebellar artery with the upper posterior inferior cerebellar artery at the V4 segment (white arrow) and lower posterior inferior cerebellar artery at the V3 segment (black arrow).

Left VA dissection and left medullary infarction were suspected because of occipital pain at the onset of the stroke. Oral aspirin therapy was initiated at a daily dose of 100 mg, and occipital pain gradually improved. On day six, cerebral angiography demonstrated recanalization of the left VA, and no abnormal findings suggestive of VA dissection were observed. It further revealed the double origin of the PICA (DOPICA) with the upper PICA (V4 segment) and lower PICA (V3 segment), and the lower PICA was more developed than the upper PICA (Figure 2).

**Figure 3:**
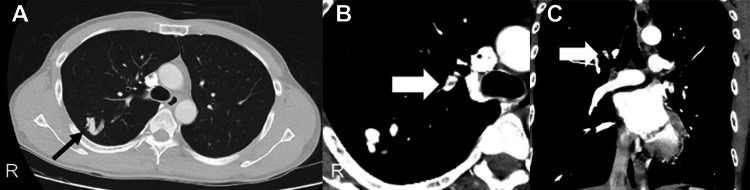
Computed tomography scan of the chest. (A) A pulmonary arteriovenous fistula with a dilated draining vein is observed on the right S2 (black arrow). The contrast-enhanced computed tomography scan shows the thrombus (white arrow) in the feeding artery. (B) Axial view. (C) Coronal view.

Thus, no cerebellar infarction occurred in the left PICA territory at the time of VA occlusion in the V4 segment. Autoantibody, coagulation (including protein S and C activity), and antiphospholipid antibody levels were within normal ranges. Holter ECG revealed no atrial fibrillation, and there were no abnormal findings on transthoracic echocardiography. On day seven, whole-body contrast-enhanced CT was performed to identify the underlying cause. In the S2 segment of the right lung, an arteriovenous fistula with a dilated draining vein was found and a suspected thrombus in the feeding artery was observed (Figure 3).

**Figure 4:**
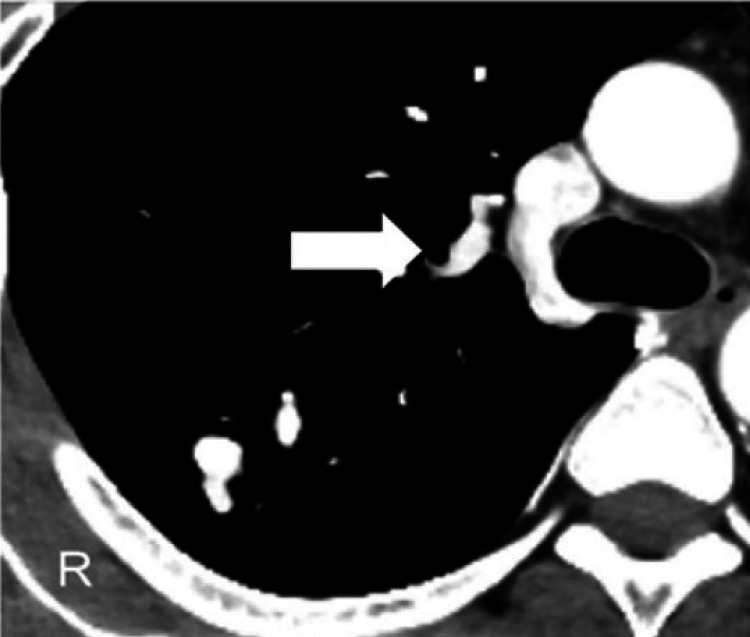
Contrast-enhanced computed tomography scan of the chest on day 64. A follow-up computed tomography confirms the disappearance of the thrombus (white arrow).

Therefore, a paradoxical cerebral embolism caused by PAVF was diagnosed. Although there were no findings suggestive of thrombosis in the veins of the lower limbs, anticoagulant therapy was necessary to prevent embolism recurrence. For the treatment of PAVF, thoracoscopic partial lung resection was considered because the fistula in the distal location was single and small. As PAVF with a thrombus in the feeding artery has the risk of cerebral embolism during surgical intervention, surgical resection was planned after confirming the disappearance of the thrombus under anticoagulant therapy. Aspirin was discontinued, and warfarin with continuous drip infusion of heparin was initiated. In addition to recurrent epistaxis and the finding of PAVF, the physical examination revealed peripheral vasodilatation of the tongue, and HHT was diagnosed. His neurological symptoms gradually improved, and he was discharged on day 21. Follow-up CT confirmed the disappearance of the thrombus in the feeding artery (Figure 4).

Thoracoscopic partial lung resection was eventually performed five months after the onset. The patient is currently on warfarin while the origin of the thrombus remains unknown. He displayed progression without the recurrence of cerebral ischemia. Informed consent was obtained from the patient for the publication of this case report.

## Discussion

Paradoxical cerebral embolism is commonly caused by PAVF; however, PAVF accompanied by Wallenberg syndrome has not yet been reported in the literature. Our patient was initially suspected to have had VA dissection because of the presence of sudden occipital pain and Wallenberg syndrome. Lebedeva et al. reported the incidence of headache at the onset of ischemic stroke in 82 (14.9%) of 550 patients, and these headaches were associated with posterior circulation stroke [5]. They argued that the emboli can cause aseptic inflammation in the vascular endothelium, thereby resulting in the release of cytokines that dilate the arteries and irritate perivascular nerve endings, causing nociception. Similar to the present case, Takeda et al. reported paradoxical cerebral embolism of PAVF in the posterior circulation with a headache [6]. In their case, the patient suddenly developed occipital pain with visual field defects and reported right posterior cerebral artery branch occlusion along with cerebral infarction in the right temporal lobe. Interestingly, in the present case, infarction occurred only in the perforator area of the brainstem, and the imaging findings were similar to Wallenberg syndrome caused by VA dissection. The underlying mechanism, wherein the infarct was observed in the perforator area, was attributed to the fact that the left PICA was not occluded because of the existence of DOPICA. This anatomical anomaly is a PICA variant that manifests as rarely as ­in 0.­­36­­-­1.45% of cases [7]. A previous study reported DOPICA to be the embryologic persistence of the normal anastomosis between the lateral spinal artery and the PICA. The lower PICA is the development of an embryonic lateral spinal artery, and the upper PICA, as true PICA, has the perforators for the medulla oblongata [8]. In the present case, the VA was occluded at the V4 segment, while the blood supply from the developed lower PICA (V3 segment) prevented cerebellar infarction of the PICA territory; nevertheless, brain stem infarction occurred due to the occlusion of the perforators from the upper PICA or the VA around the upper PICA.

There are few reports on malformations with thrombus in the feeding artery, and due to the risk of intraoperative ischemic complications caused by the thrombus in this type of PAVF, a standard treatment strategy has not been established [9,10]. Serra et al. administered anticoagulant therapy for one week to a patient with PAVF and confirmed the disappearance of the thrombus in the feeding artery, and the embolization was performed without the recurrence of cerebral infarction [10]. In this case, after the disappearance of the thrombus in the feeding artery upon warfarin treatment, thoracoscopic partial lung resection was successfully performed, which resulted in a complete cure.

This is an extremely rare case of acute onset of severe headache with Wallenberg syndrome mimicking VA dissection, which was caused by paradoxical cerebral embolism of a PAVF-accompanied thrombus in the feeding artery.

## Conclusions

PAVF is often accompanied by the autosomal dominant inheritance of HHT. If left untreated, this condition is potentially fatal and, therefore, warrants considerable attention. Even in patients with headache and Wallenberg syndrome who are suspected to have undergone arterial dissection, when clear recanalization is observed, cerebral embolism, including paradoxical cerebral embolism of the PAVF, should be considered in the differential diagnosis.

## References

[1] Gossage JR, Kanj G (1998). Pulmonary arteriovenous malformations. A state of the art review. Am J Respir Crit Care Med.

[2] White RI Jr, Lynch-Nyhan A, Terry P (1988). Pulmonary arteriovenous malformations: techniques and long-term outcome of embolotherapy. Radiology.

[3] Muri JW (1955). Arteriovenous aneurysm of the lung. Am J Surg.

[4] Lacombe P, Lacout A, Marcy PY (2013). Diagnosis and treatment of pulmonary arteriovenous malformations in hereditary hemorrhagic telangiectasia: an overview. Diagn Interv Imaging.

[5] Lebedeva ER, Ushenin AV, Gurary NM, Tsypushkina TS, Gilev DV, Kislyak NV, Olesen J (2021). Headache at onset of first-ever ischemic stroke: clinical characteristics and predictors. Eur J Neurol.

[6] Takeda J, Todo K, Yamamoto S, Yamagami H, Kawamoto M, Kohara N (2012). [Paradoxical brain embolism mediated through a pulmonary arteriovenous malformation with hereditary hemorrhagic telangiectasia in a Japanese patient]. Rinsho Shinkeigaku.

[7] Lesley WS, Dalsania HJ (2004). Double origin of the posterior inferior cerebellar artery. AJNR Am J Neuroradiol.

[8] Lasjaunias P, Vallee B, Person H, Ter Brugge K, Chiu M (1985). The lateral spinal artery of the upper cervical spinal cord. Anatomy, normal variations, and angiographic aspects. J Neurosurg.

[9] Cha JG, Hong J (2022). Concurrent pulmonary arteriovenous malformation and pulmonary embolism causing stroke: a therapeutic dilemma. CVIR Endovasc.

[10] Serra MM, Ferreyro BL, Peralta O (2015). Huge pulmonary arteriovenous malformation, venous thromboembolism and anticoagulation treatment in a patient with hereditary hemorrhagic telangiectasia. Intern Med.

